# Disseminated angiostrongylosis with involvement of the central nervous system as a cause of sudden death in a dog in Germany

**DOI:** 10.1186/s12917-026-05574-w

**Published:** 2026-05-22

**Authors:** Martin Dembowski, Kristin Pütsch, Cora Delling, Christian Bauer, Katja Kalenyak, Florian Hansmann

**Affiliations:** 1 Institute of Veterinary Pathology, Faculty of Veterinary Medicine, Leipzig University, Leipzig, Germany; 2 Institute of Parasitology, Faculty of Veterinary Medicine, Leipzig University, Leipzig, Germany; 3 Institute of Parasitology, Justus Liebig University Giessen, Giessen, Germany; 4 Department for Small Animals, Faculty of Veterinary Medicine, Leipzig University, Leipzig, Germany

**Keywords:** *Angiostrongylus vasorum*, Dog, Disseminated angiostrongylosis, Germany

## Abstract

**Background:**

The metastrongyloid nematode *Angiostrongylus vasorum*, known as “French Heartworm”, is an emerging concern in canine health in Europe. Foxes are considered the main reservoir hosts, and affected carnivores predominantly show cardiovascular and respiratory signs. However, a disseminated course of infection can have diverse and severe consequences, including fatal outcomes.

**Case presentation:**

A six-year-old male Labrador Retriever was presented to the clinic with progressive episodes of stupor lasting a few seconds and dyspnea. Radiographic examination revealed multifocal pulmonary consolidation. In addition, a prolonged blood clotting time was detected. The dog died of acute cardiovascular failure. Histological examination revealed a multifocal, moderate granulomatous pneumonia with numerous intralesional nematode first stage larvae and eggs and interstitial fibrosis in the lungs as well as nematode larvae in the kidneys, brain, spinal cord and myocardium. In addition to morphology, the identity of the nematode larvae as *A. vasorum* was confirmed by PCR.

**Conclusions:**

Several case reports and studies show that the endemic regions of *A. vasorum* are expanding, and consequently the number of affected countries is increasing. Angiostrongylosis, particularly the disseminated form, remains an underestimated cause of death in dogs and should be taken into consideration in dogs with an unknown cause of disease, especially when cardiovascular or respiratory symptoms and/or neurological signs are present.

**Supplementary Information:**

The online version contains supplementary material available at 10.1186/s12917-026-05574-w.

## Background

Increased travel by humans and dogs, urbanization of wildlife resulting in a growing interface between wild and domestic animals, and climate change all contribute to the geographical spread of existing canine pathogens, as well as to the emergence of infectious agents—particularly parasitic species—previously unknown in a given locality. In Central Europe, these include the helminths Echinococcus *multilocularis*, *Dirofilaria immitis*, *Dirofilaria repens*, and *Angiostrongylus vasorum*. *E. multilocularis* and *Dirofilaria* spp. are of zoonotic relevance, while *Dirofilaria* spp. and *A. vasorum* pose significant health threats, particularly to dogs and wild carnivores [1–5].

*A. vasorum* has an indirect life cycle [6]. In Europe, the red fox is its main reservoir host, harboring the adult worm stages as a definitive host in the pulmonary arteries and right heart. Other wild carnivores—including wolves, golden jackals, raccoon dogs, badgers, and weasels—as well as domestic dogs, also serve as definitive hosts. These hosts excrete larvae in their feces, which are subsequently ingested by gastropods, such as slugs (e.g., *Arion* spp.), acting as intermediate hosts [7, 8]. Frogs may function as paratenic hosts [9]. Within the intermediate hosts, the larvae develop into infective stages and are subsequently acquired by the definitive host through ingestion.

Until the 1970s, the geographic distribution of *A. vasorum* was restricted to a few specific regions, including France (hence the name “French heartworm”). Since then, both the detection frequency and geographic range of *A. vasorum* in foxes and dogs have continuously increased [10–12]. For instance, the prevalence of angiostrongylosis in red foxes in Switzerland was 21% in 2012 but reached 82% only five years later [13]. In Germany, the rate of fecal excretion of *A. vasorum* larvae in dogs increased significantly from 1% during 2004–2006 to 3.1% during 2015–2017 [14]. Available data from Germany reveal a distinct spatial distribution of this nematode species: studies of red fox carcasses suggested an eastward spread of the parasite, with prevalences of 27%, 19%, and 8% in Rhineland-Palatinate, Hesse, and Thuringia, respectively [15]. Dogs in southwestern and northeastern Germany were significantly more likely to test positive by coproscopy than dogs in other parts of the country [16]. However, the occurrence of *A. vasorum* in dogs in Saxony has so far been documented in only a single coproscopically confirmed case [17].

Two main clinical syndromes are typically observed in association with *A. vasorum* infection: respiratory disease resulting from an inflammatory response to eggs and migrating larvae, and haemorrhagic diathesis characterized by localized or generalized bleeding [18, 19]. Consequently, affected animals may exhibit a combination of cardiorespiratory (coughing and/or dyspnoea), coagulopathic (haemorrhagic diathesis), and other clinical signs, including exercise intolerance, lethargy, weakness, and collapse [18]. The migration of *A. vasorum* larvae through the alveolar walls can lead to focal haemorrhages, resulting in thrombosis and occlusion of pulmonary arteries and triggering granulomatous inflammation [6]. Most animals develop respiratory signs and heart failure due to elevated pulmonary blood pressure, which is associated with cardiac dilation, systemic congestion, and ascites [6]. Thoracic radiographs typically reveal a characteristic mottled alveolar–interstitial pattern. In more severe cases, pleural fissure lines may be visible, suggesting pleural effusion [18]. In addition, a consumptive coagulopathy characterized by the formation of microthrombi and haemorrhagic diathesis may develop [6]. It has been hypothesized that these abnormalities are caused by vascular damage resulting from larval migration and increased consumption of clotting factors, likely induced by antigenic properties of the parasite [19–21]. Dogs affected by haemorrhagic diathesis typically present with varying degrees of anaemia, thrombocytopenia, and prolongation of prothrombin time and/or activated partial thromboplastin time [18].

Systemic dissemination of *A. vasorum* larvae to organs including the central nervous system (CNS), myocardium, liver, spleen, and eye has been repeatedly reported in dogs with pulmonary angiostrongylosis [22]. Neurological manifestations associated with disseminated angiostrongylosis depend on the localization of lesions within the CNS and may include seizures, cranial nerve deficits, vestibular dysfunction, proprioceptive deficits, ataxia, and paraplegia [23–26]. However, it is important to note that the boundaries between the aforementioned syndromes are not clearly delineated and may partially overlap [18, 19]. Furthermore, the severity of clinical signs does not necessarily correlate with the intensity of infection [27].

Therefore, even in cases with unclear cardiorespiratory symptoms, angiostrongylosis should be considered. The present report describes a case of disseminated angiostrongylosis in a dog from Saxony, Germany with a fatal neurological outcome.

## Case presentation

### Clinical findings and treatment

A six-year-old male Labrador Retriever, weighing 39.5 kg, from Chemnitz (Saxony, Germany), was presented to the emergency service of the Department for Small Animals at Leipzig University with progressive episodes of stupor lasting several seconds and respiratory distress. The dog had spent its entire life in Germany. On presentation, it was in poor general condition and exhibited recurrent brief episodes of unconsciousness. Clinical examination revealed pale pink mucous membranes, a capillary refill time of less than 2 s, a heart rate of 104 beats per minute with a strong pulse, and a respiratory rate of 88 breaths per minute. Cardiac auscultation was unremarkable, whereas pulmonary auscultation revealed laboured breathing with increased respiratory sounds. Body temperature was 38.4 °C. The peripheral lymph nodes were non-painful and normal in size.

The dog was immediately treated with oxygen supplementation via nasal cannula and intravenous fluid therapy, adjusted according to volume and hydration status, using a crystalloid solution (Ringer’s acetate, B. Braun, Melsungen, Germany). In addition, the dog received one unit (300 ml) of fresh frozen plasma, vitamin K₁ (Vitamivet K1, DOMES PHARMA, Pont-du-Château, France; initially 2.5 mg/kg PO), and amoxicillin/clavulanic acid (AmoxClav HEXAL^®^ i.v. 1000/200 mg, Hexal AG, Holzkirchen, Germany; 20 mg/kg IV, TID). Despite treatment, the dog suffered cardiac arrest shortly after hospital admission.

### Laboratory analysis

Complete blood count measured using a ProCyte Dx haematology analyser (IDEXX Laboratories, Hoofddorp, Netherlands) revealed a mild regenerative anaemia and mild thrombocytopenia (Table 1).

**Table 1:** Complete blood count

Parameter	Result	Reference interval
Red blood cells (M/µL)	4.30	5.65–8.87
Hematocrit (%)	31.1	37.3–61.7
Hemoglobin (g/dL)	11.0	13.1–20.5
MCV (fL)	72.3	61.6–73.5
MCH (pg)	25.6	21.2–25.9
MCHC (g/dL)	35.4	32.0–37.9
RDW (%)	15.8	13.6–21.7
Reticulocytes (%) ETIC	4.9	
Reticulocytes (K/µL)	212.0	10.0–110.0
Reticulocyte hemoglobin (pg)	28.1	22.3–29.6
White blood cells (K/µL)	13.53	5.05–16.76
Neutrophils (%)	70.4	
Lymphocytes (%)	15.4	
Monocytes (%)	10.6	
Eosinophils (%)	3.5	
Basophils (%)	0.1	
Neutrophils (K/µL)	9.51	2.95–11.64
Lymphocytes (K/µL)	2.09	1.05–5.10
Monocytes (K/µL)	1.44	0.16–1.12
Eosinophils (K/µL)	0.48	0.06–1.23
Basophils (K/µL)	0.01	0.00–0.10
Platelets (K/µL)	96	148–484
MPV (fL)	12.2	8.7–13.2
PDW (fL)	12.3	9.1–19.4
Plateletcrit (%)	0.12	0.14–0.46

*MCV* mean corpuscular volume, *MCH*   mean corpuscular hemoglobin, *MCHC*  mean corpuscular hemoglobin concentration, *RDW*  red cell distribution width, *MPV* mean platelet volume, *PDW*  platelet distribution width

Coagulation tests were performed using a Coag Dx™ analyzer (IDEXX Laboratories, Hoofddorp, Netherlands). Both prothrombin time (PT) and activated partial thromboplastin time (aPTT) were markedly prolonged (PT: 47 s, reference interval 11–17 s; aPTT: 300 s, reference interval 72–102 s). The serum biochemistry profile was unremarkable (Supplementary Table 1).

### Imaging

**Fig. 1:**
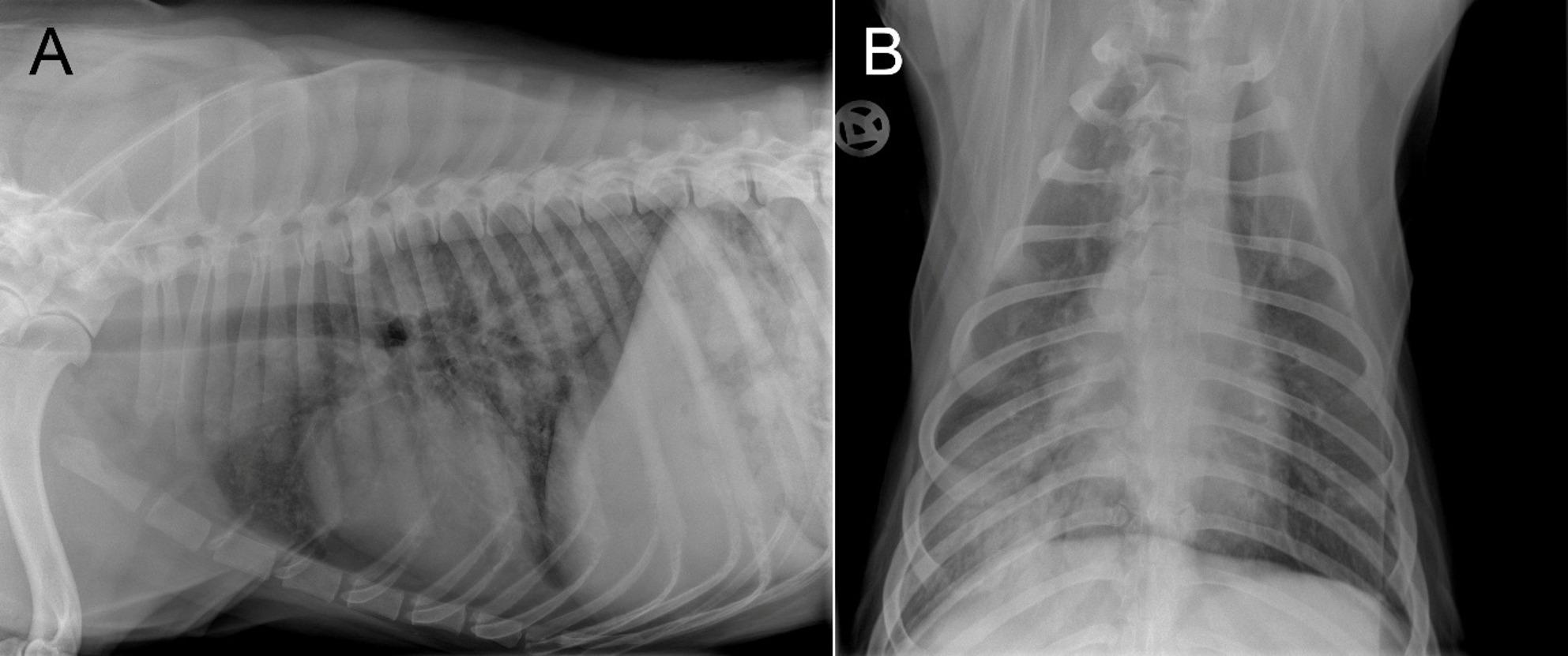
Thoracic radiographs, in laterolateral (**A**) and ventrodorsal (**B**) view of the dog, showing a diffuse mixed bronchial and unstructured interstitial lung pattern with additional alveolar opacity predominantly localised to the right caudal lung lobe

Two-view thoracic radiographs (DigitalDiagnost, Philips GmbH, Hamburg, Germany) revealed diffuse, hazy, unstructured pulmonary opacities with a marked alveolar pattern predominantly affecting the right caudal lung lobe (Fig. 1).

### Post-mortem findings

**Fig. 2:**
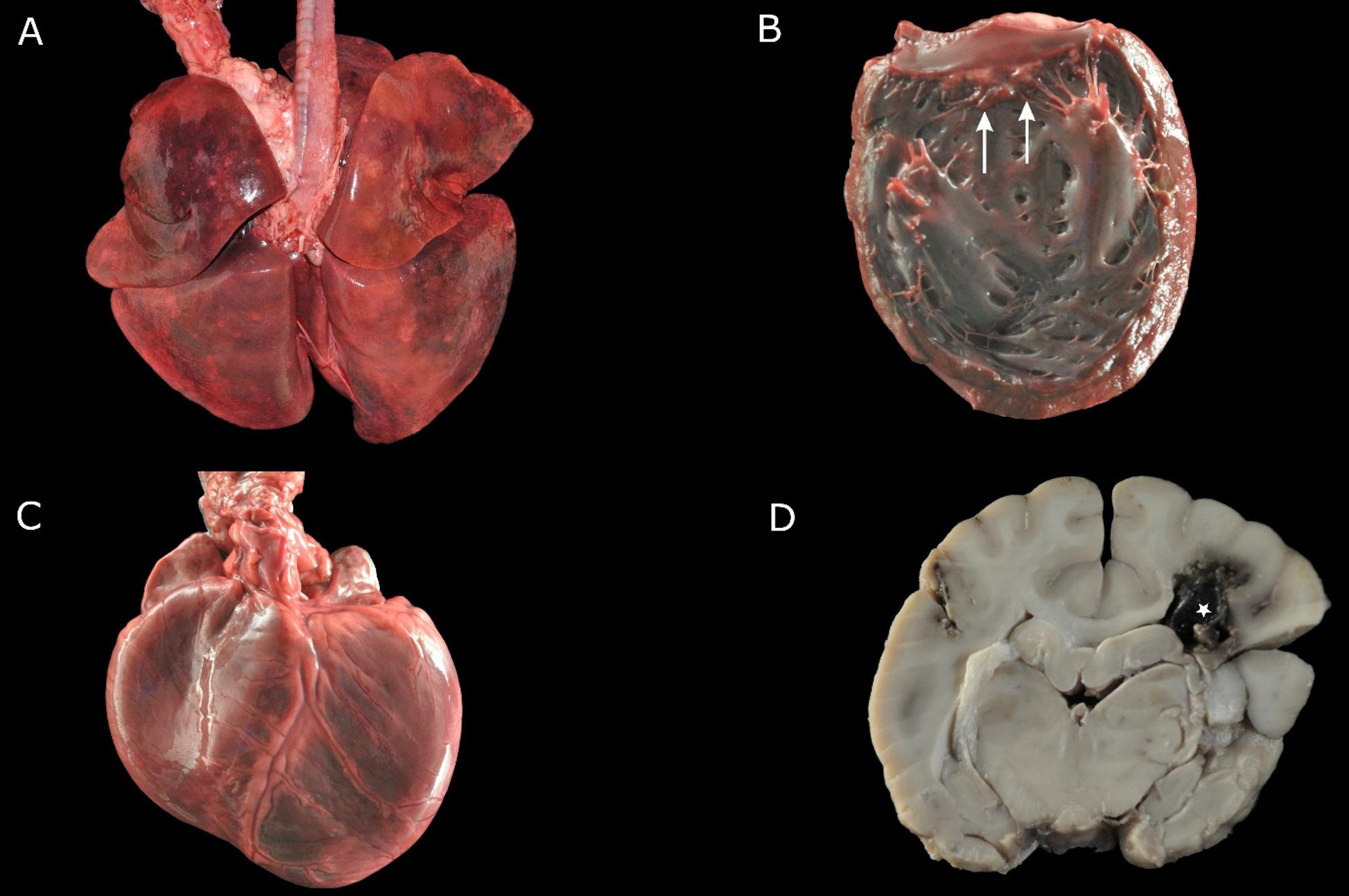
Gross findings of the lungs, heart and brain of the dog. **A** The lungs were poorly retracted and showed a firm consistency with multifocal consolidated areas. **B**, **C** The heart revealed a multifocal endocardiosis of the mitral valve (arrows) and dilation of both ventricles. **D** In the brain, multifocal haemorrhages (★), accentuated in the white matter of the cerebrum, were detected

Gross examination revealed firm to rubbery, poorly retracted, dark red lungs with multifocal small, consolidated areas of 1 to 3 cm in diameter (Fig. 2A). The heart showed multifocal endocardiosis of the mitral valve (Fig. 2B), associated with a mild to moderate dilation of both ventricles (Fig. 2C). In the brain, one large as well as multiple smaller haemorrhages, accentuated in the cerebrum (Fig. 2D), were detected.

**Fig. 3:**
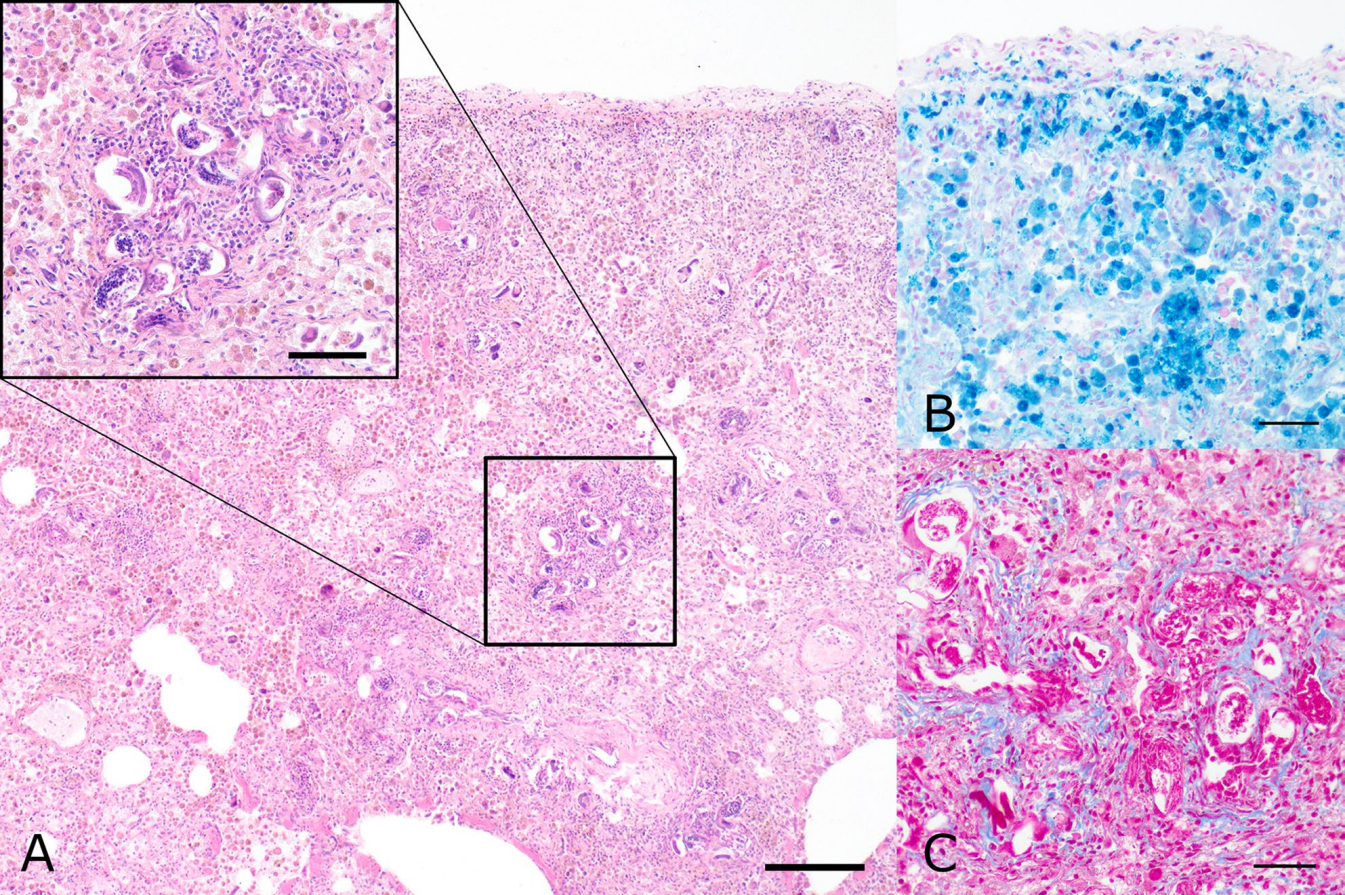
Histological findings of the lungs from the dog. **A** Numerous sections of nematode larvae and eggs were located in the lung parenchyma (Hematoxylin and eosin staining). Scale bars indicate 200 μm in the overview and 100 μm in the inset. **B** Quincke staining visualized iron storage in alveolar macrophages. Scale bar indicates 50 μm. **C** Azan staining identified multifocal interstitial fibrosis of the lungs. Scale bar indicates 50 μm

Tissue samples for histological and histochemical examination were collected from the brain, spinal cord, lung, liver, spleen, kidneys, heart, eyes, thyroid, stomach, and both the small and large intestines. The samples were fixed in 10% neutral buffered formalin overnight, followed by dehydration and embedding in paraffin wax. Section 2 μm thick were cut and stained with HE; additionally, histochemical special stains including Azan and Quincke stains were performed [28]. Light microscopic examination of the lungs revealed a multifocal granulomatous pneumonia with multinucleated giant cells and numerous intralesional nematode larvae and eggs (Fig. 3).

**Fig. 4:**
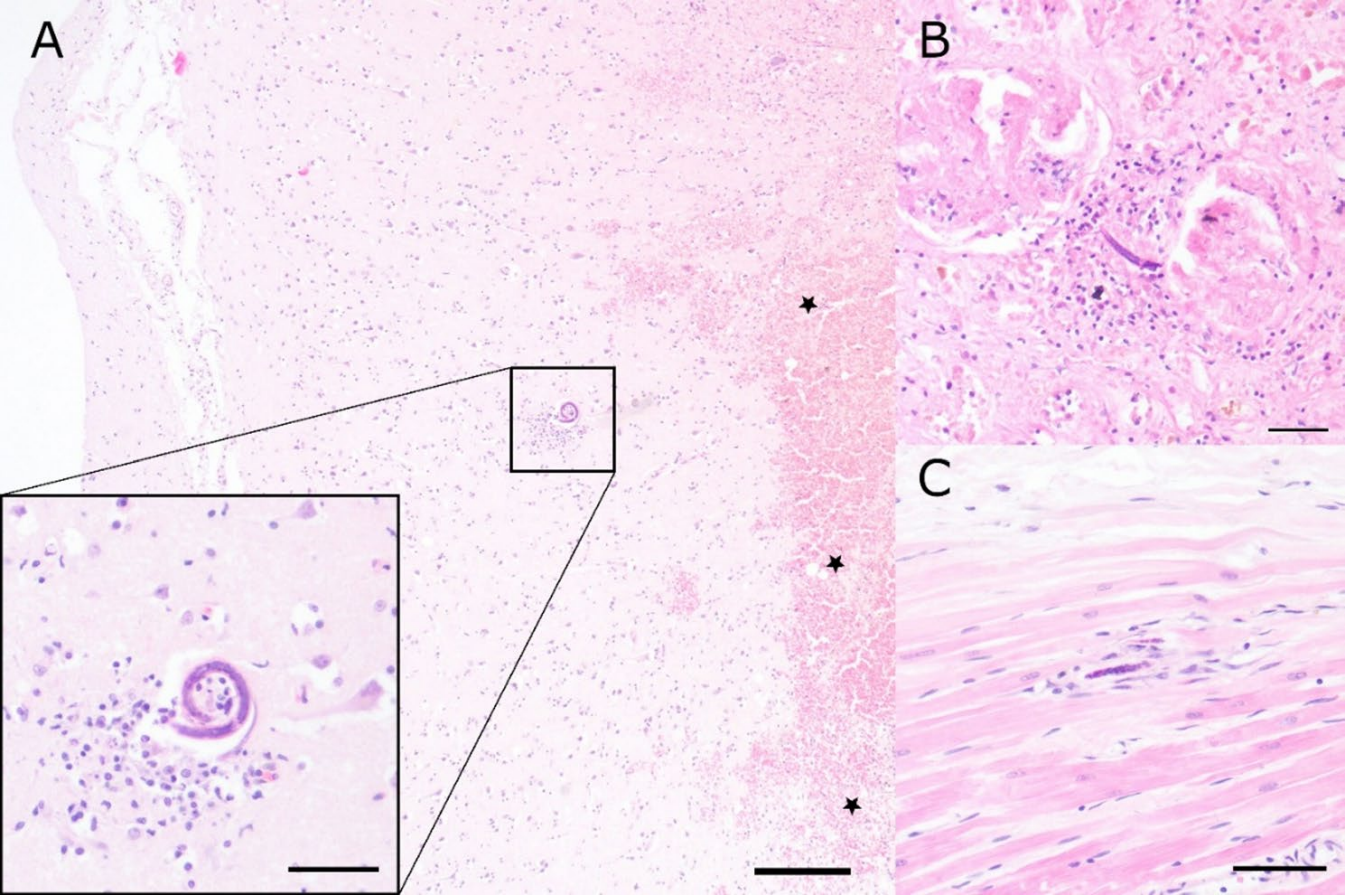
Histological findings of the brain, kidneys and heart from the dog. **A** Within the brain parenchyma, multifocal granulomatous inflammation with intravascular nematode larvae and multifocal, moderate to severe acute haemorrhages (★) were observed. Scale bars indicate 200 μm in the overview and 50 μm in the inset. **B**, **C** Multifocal intravascular nematode larvae were detected in the kidneys (**B**) and myocardium (**C**). Scale bars indicate 50 μm

Nematode larvae measured about 20 μm in diameter and showed an eosinophilic cuticle, hypodermis, coelomyarian musculature, a pseudocoelom and an intestinal tract. Eggs were thin walled, measured about 50 × 80 μm and contained either a morula or a larva. Nematode larvae were also found in the kidneys, brain, spinal cord and myocardium (Fig. 4).

In association with the nematode stages, haemorrhages, granulomatous inflammation, as well as granulation tissue or fibrosis were detected. The morphological characteristics and location of larvae and eggs within the lung parenchyma, together with the observed histological alterations and the presence of larvae in various extraintestinal tissues, led to a suspected diagnosis of disseminated *A. vasorum* infection.

### Molecular diagnostics

DNA extraction from frozen lung tissue was performed using the NucleoSpin Tissue kit (Macherey-Nagel, Düren, Germany) according to the manufacturer’s instructions. A PCR targeting a fragment of approximately 1,700 bp of the nuclear 18 S rRNA gene was performed using primers NC18SF1 and NC5BR and the thermal cycling protocol as previously described [29]. Three microlitres of DNA template were used per reaction. The PCR mixture contained 0.5 µl of each primer, 0.8 µl of dNTPs, 0.1 U of DreamTaq DNA polymerase (Thermo Fisher Scientific, Dreieich, Germany), 2.5 µl of DreamTaq™ Green Buffer (10×; Thermo Fisher Scientific, Dreieich, Germany), and nuclease-free water to a final volume of 25 µl. PCR products were subsequently sequenced in both directions by a commercial company (Microsynth Seqlab, Göttingen, Germany). Comparison of the consensus sequence (NCBI Nucleotide PX977118) with sequences available in the GenBank database using BLASTn analysis revealed a sequence similarity of 99.75% to published A. vasorum sequences (AJ920365.1, EF514916.1), thereby confirming the morphological diagnosis of A. vasorum as the aetiologic agent in this case.

## Discussion and conclusions

The present case represents an autochthonous case of disseminated *A. vasorum* infection involving the CNS in a dog in Germany. The animal lived in Saxony—a region where the prevalence of this parasite has hitherto been low [16, 17]—and, according to its medical history, had never travelled abroad. However, it cannot be entirely excluded that the dog acquired the infection in another region of Germany where the infection is known to be more widespread [16].

*A. vasorum* infection was characterized by atypical larval dissemination to the brain, resulting in progressive episodes of stupor lasting few seconds as the predominant clinical sign. This presentation can be misdiagnosed as idiopathic epilepsy or other inflammatory CNS diseases, such as meningoencephalitis of unknown origin, including granulomatous meningoencephalitis, rabies, or bacterial, viral, or protozoal encephalitis. In general, a broad spectrum of inflammatory and non-inflammatory aetiologies should be considered in the differential diagnosis of such respiratory and neurological signs.

As described above, the parasites’ different sites of infection can lead to a range of clinical signs. In this context, the marked inflammatory changes and presence of parasites in the lungs are very likely associated with the observed respiratory signs. Likewise, the severe haemorrhages in the CNS, together with the detection of larvae in this location, are likely responsible for the observed neurological signs. Moreover, the prolonged coagulation times may also have contributed to the cerebral haemorrhages and, consequently, to the neurological signs. Nevertheless, it is also possible that the neurological signs were partly attributable to hypoxia secondary to heart failure.

Intravital diagnosis can be made by detecting larvae in faeces (Baermann funnel technique) or in bronchoalveolar lavage fluid, although both methods have relatively low sensitivity. Serological detection of circulating *A. vasorum* antigen or specific antibodies is also possible [30]. Furthermore, post mortem, in addition to histopathological examination and molecular methods can be used for diagnosis [27].

Based on the morphological appearance of the parasite in histopathological sections, several etiological differential diagnoses must be considered, including migrating larvae of *Toxocara canis*, hookworm species, *Strongyloides stercoralis*, and *Baylisascaris procyonis*. However, these nematodes can be excluded based on the presence of eggs within extraintestinal parenchyma [31]. Furthermore, fatal extraintestinal larval infections with Toxocara canis are observed primarily in puppies and young dogs [32], whereas disseminated infection with Strongyloides stercoralis is typically encountered in immunosuppressed dogs [33, 34]. *Baylisascaris* larvae exhibit a significantly larger diameter (approximately 60 μm) [35, 36] than that observed in the present case (approximately 20 μm). Additionally, infection with *Filaroides hirthi* must be considered as differential diagnosis. Although adults, larvae, and eggs of this nematode can be detected within lung tissue, they usually do not induce marked tissue reactions; however, in immunocompromised dogs, inflammatory changes with severe clinical consequences may occur. Notably, there is no extraintestinal dissemination of this lungworm species [37, 38].

In this context, it is also pertinent to mention other *Angiostrongylus* species, such as the *A. cantonensis* (the “rat lungworm”), which has been detected in dogs and has a zoonotic potential [39–42]. Formerly not-endemic in Europe, increasing numbers of *A. cantonensis* cases have been reported on the Canary and Balearic Islands, with the potential to spread to mainland Europe [5, 43, 44].

In conclusion, *A. vasorum* infections in dogs can present with a wide range of clinical manifestations. In disseminated cases, cardiopulmonary symptoms may be less prominent, while neurological signs dominate the clinical picture, as observed in the present case.

## Supplementary Information

Supplementary Material 1.

## Data Availability

All data obtained or analysed as part of this study are included in this article.

## Abbreviations

CNS: Central nervous system
DNA: Deoxyribonucleic acid
HE: Hematoxylin and eosin
PCR: Polymerase chain reaction
