# Supplementary material for: Disseminated angiostrongylosis with involvement of the central nervous system as a cause of sudden death in a dog in Germany
Source: BMC Vet Res. 2026 May 22;22:302. doi: 10.1186/s12917-026-05574-w (PMC13198027; doi:10.1186/s12917-026-05574-w)
Supplement: Supplementary file 1 — Supplementary Material 1. [file 12917_2026_5574_MOESM1_ESM.pdf]

Supplementary table 1: Biochemistry profile (measured by the referring veterinarian in-house on IDEXX Catalyst One just prior to referral to the Small Animal Clinic at Leipzig University)

Parameter	Result	Reference interval
Glucose (mg/dL)	130	74 - 143
SDMA (µg/dL)	10	0 – 14
Creatinine (mg/dL)	1.0	0.5 - 1.8
Urea (mg/dL)	11	7 – 27
BUN/CREA	11	
Phosphorus (mg/dL)	4.7	2.5 - 6.8
Calcium (mg/dL)	9.6	7.9 - 12.0
Total protein (g/dL)	7.4	5.2 - 8.2
Albumin (g/dL)	3.1	2.3 - 4.0
Globulin (g/dL)	4.2	2.5 - 4.5
Albumin/Globulin	0.7	
Alanine transferase (U/L)	51	10 - 125
Alkaline phosphatase (U/L)	46	23 - 212
Gamma-glutamyltransferase (U/L)	1	0 - 11
Total bilirubin (mg/dL)	< 0.1	0.0 - 0.9
Cholesterol (mg/dL)	86	110 - 320
Amylase (U/L)	658	500 - 1500
Lipase (U/L)	273	200 - 1800
Sodium (mmol/L)	153	144 - 160
Potassium (mmol/L)	4.5	3.5 - 5.8
Sodium/Potassium	34	
Chloride (mmol/L)	107	109 - 122
Osmolality calculated (mmol/kg)	303	
Total T4 (µg/dL)	2.2	1.0 - 4.0

SDMA = symmetric dimethylarginine
